# Minocycline attenuation of rat corpus callosum abnormality mediated by low-dose lipopolysaccharide-induced microglia activation

**DOI:** 10.1186/s12974-021-02142-x

**Published:** 2021-04-26

**Authors:** Jingdong Zhang, Michael Boska, Ya Zheng, Jianuo Liu, Howard S. Fox, Huangui Xiong

**Affiliations:** 1 Department of Pharmacology & Experimental Neuroscience, University of Nebraska Medical Center, Omaha, NE 68198 USA; 2 Present Address: Department of Anesthesiology, University of Cincinnati College of Medicine, Cincinnati, OH 45267 USA; 3 Department of Radiology, University of Nebraska Medical Center, Omaha, NE 68198 USA; 4 Present address: Department of Rehabilitation Medicine, Tongji Hospital Affiliated to Tongji University School of Medicine, Shanghai, 200065 China; 5 Department of Neurological Sciences, University of Nebraska Medical Center, Omaha, NE 68198 USA

**Keywords:** Lipopolysaccharide, Microglia activation, Neuroinflammation, Corpus callosum dysfunction, Minocycline

## Abstract

**Background:**

Microglia are resident innate immune cells in the brain, and activation of these myeloid cells results in secretion of a variety of pro-inflammatory molecules, leading to the development of neurodegenerative disorders. Lipopolysaccharide (LPS) is a widely used experimental stimulant in microglia activation. We have previously shown that LPS produced microglia activation and evoked detectable functional abnormalities in rat corpus callosum (CC) in vitro. Here, we further validated the effects of low-dose LPS-induced microglia activation and resultant white matter abnormality in the CC in an animal model and examined its attenuation by an anti-inflammatory agent minocycline.

**Methods:**

Twenty-four SD rats were divided randomly into three groups and intra-peritoneally injected daily with saline, LPS, and LPS + minocycline, respectively. All animals were subject to MRI tests 6 days post-injection. The animals were then sacrificed to harvest the CC tissues for electrophysiology, western blotting, and immunocytochemistry. One-way ANOVA with Tukey’s post-test of all pair of columns was employed statistical analyses.

**Results:**

Systemic administration of LPS produced microglial activation in the CC as illustrated by Iba-1 immunofluorescent staining. We observed that a large number of Iba-1-positive microglial cells were hyper-ramified with hypertrophic somata or even amoeba like in the LPS-treated animals, and such changes were significantly reduced by co-administration of minocycline. Electrophysiological recordings of axonal compound action potential (CAP) in the brain slices contained the CC revealed an impairment on the CC functionality as detected by a reduction in CAP magnitude. Such an impairment was supported by a reduction of fast axonal transportation evidenced by β-amyloid precursor protein accumulation. These alterations were attenuated by minocycline, demonstrating minocycline reduction of microglia-mediated interruption of white matter integrity and function in the CC.

**Conclusions:**

Systemic administration of LPS produced microglia activation in the CC and resultant functional abnormalities that were attenuated by an anti-inflammatory agent minocycline.

## Background

Microglia are resident innate immune cells in the central nervous system (CNS) [1, 2] and have diverse functions in the brain under normal and disease conditions. They are involved in the immunity, neurogenesis, synaptogenesis, neurotrophic support, removal of cellular/tissue debris, and maintaining CNS homeostasis [3]. Microglia appear as ramified cells in the resting condition and change their shape to an amoeboidic form once activated. Ample evidence indicate that microglia are one of the major cell types involved in the inflammatory responses in the CNS. The activated microglia release a variety of pro-inflammatory molecules including, but not limited to, chemokines, cytokines, cyclooxygenase-2, inducible nitric oxide synthase, and nitric oxide. These substances provoke inflammatory responses and brain tissue damage that contribute to the pathogenesis of neurodegenerative disorders including Alzheimer’s disease [4–8], Parkinson’s disease [9, 10], and human immunodeficiency virus type 1(HIV-1)-associated neurocognitive disorders [11, 12].

Many bioactive substances and factors can induce microglial cell activation. Lipopolysaccharide (LPS, a bacterial endotoxin) is the most commonly used pro-inflammatory stimulus for microglia both in vitro and in vivo. It is well-established that LPS-associated neuropathology stems from the microglia activation and resultant release of cytokines and inflammatory mediators [13–16]. We have previously shown that LPS, at low doses, produced microglia activation in rat corpus callosum (CC) in vitro and evoked detectable functional abnormalities on white matter tracts in the absence of overt axonal injury, hypoxia, and trauma that could be detrimental to the white matter [17]. Those findings imply that suppression of microglia activation in the CC by anti-inflammatory reagents may have a therapeutic benefit in protecting white matter tracts from inflammatory assault.

Ample evidence indicate that minocycline (7-dimethylamino-6-dimethyl-6-deoxytetracycline), a second-generation semisynthetic tetracycline analog, has a neuroprotective capacity in various animal studies. It is a highly lipophilic molecule that can easily penetrate the blood-brain barrier [18], thus promoting its accumulation in the CNS and enabling its use in the treatment of neurodegenerative diseases [19, 20]. Furthermore, there is rapidly growing evidence indicating that minocycline may exert its neuroprotective activity through suppression of microglial activation [21], inhibition of neuroinflammatory response, and attenuation of neuronal apoptosis [22–24]. These actions appear significantly different from their antibiotic properties. Indeed, minocycline attenuated brain white matter injuries induced by intracerebral hemorrhage or cerebral ischemia through its anti-inflammatory activity [25–27]. In the present study, we attempted to validate the effects of LPS on microglia activation and consequent brain white matter abnormality observed previously in an in vitro preparation in an in vivo system and examined the protective effects of minocycline on the rat CC abnormalities induced by low-lose LPS. Our results showed that systemic administration of LPS produced microglial activation in the CC and impaired CC functionality as revealed by reduction of axonal compound action potential (CAP) magnitude and fast axonal transportation evidenced by β-amyloid precursor protein (β-APP) accumulation. These alterations were attenuated by minocycline, demonstrating minocycline reduction of microglia-mediated interruption of white matter functionality in the CC of rats.

## Materials and methods

### Animals

Twenty-four adult Sprague-Dawley rats (40–50 days old; 12 male and 12 female) were used, and they were divided into 3 groups, each with 4 male and 4 female animals. Group 1 received sterilized saline injection as a control (saline group), LPS was injected to group 2 rats (LPS group), and the third group received both LPS and minocycline injection (LPS + minocycline). All experimental protocols and animal care were carried out in accordance with the National Institutes of Health *Guide for the Care of Laboratory Animals in Research* and approved by the Institutional Animal Care and Use Committee of the University of Nebraska Medical Center. All efforts were made to minimize animal suffering and the number of animals used in this study.

### Intraperitoneal injection of saline, LPS, and LPS + minocycline

LPS (*Escherichia coli*, L2880, Sigma, St. Louise, MO) was prepared with saline at 1 mg/ml and stored at – 20 °C. Minocycline hydrochloride (M9511, Sigma) was dissolved in saline at 25 mg/ml shortly before administration. LPS of 1 mg/kg was intra-peritoneally (i.p.) injected daily in the LPS group animals, and animals in the saline group received equivalent volume of saline daily for three consecutive days. In the LPS + minocycline group, minocycline of 25 mg/kg was i.p. administrated daily 2 h before LPS injection for four consecutive days.

### Magnetic resonance imaging

**Fig. 1:**
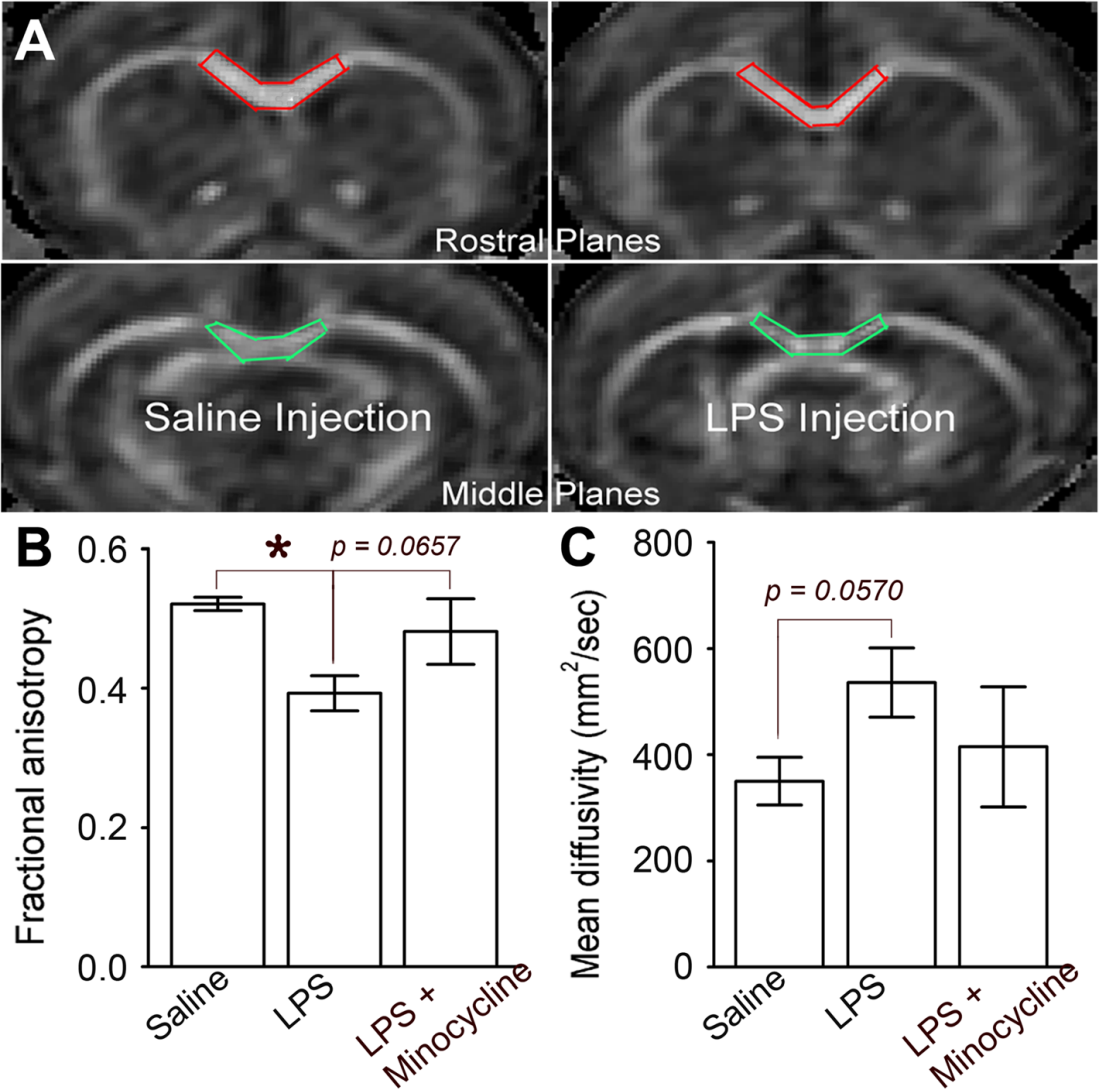
Interruption of white matter linear integrity by system LPS, obviously reversed by minocycline. **a** FA and MD values were measured and analyzed on the regions of interest in the rostral (red frame) and middle (green frame) parts of the CC body. **b** The FA values, namely linear movements of water molecules paralleling with the axons, were significantly distorted by system LPS (*N* = 8 in both saline and LPS groups; *p* < 0.05, shown as *), while minocycline reversed this distortion to an approximately significant level (*N* = 8 in both LPS and LPS + minocycline groups; *p* = 0.0657). **c** The MD values, namely water diffusion with angles to the axons, were enhanced to an almost significant level comparing to the saline group (*p* = 0.0570); however, co-delivery of minocycline seemed to invert this effect

All animals in three groups received MRI tests at the 6th day after the initial injection. The MRI tests were performed on a 7T/21-cm MR scanner (Bruker, Karlshure, Germany) equipped with Resonance Research (Billerica, MA) gradients and shims. Animals were anesthetized with a 2% isoflurane/oxygen mixture, and body temperatures were kept constant at 37 °C with a MRI-compatible heater (SAII, NY). T_2_-weighted anatomical images were acquired from 16 contiguous coronal slices with a rapid acquisition with relaxation enhancement sequence. For each animal, an average diffusion-weighted image volume including fractional anisotropy (FA) and mean diffusivity (MD) was reconstructed. To measure the diffusion indices quantitatively, regions-of-interest (ROIs; Fig. 1a, red and green frames) were drawn manually from the raw FA maps of each rat. The average FA and MD values from measurement in the ROIs were calculated and compared among the saline, LPS, and LPS + minocycline injection groups. In addition, the FA and MD values of 4 male and 4 female rats from the same group were statistically compared in each group.

### Preparation of CC slices and electrophysiology

On the day immediately after MRI tests, six animals (3 males/3 females) from each group were deeply anesthetized with isoflurane and then decapitated. The brains were quickly dissected out of the cranial cavities, placed into an ice-cold (4 °C) oxygenated artificial cerebrospinal fluid (ACSF), and cut into 500-μm (in thickness) slices as previously described [28]. Every other slice was used to perform extraction of protein for western blot. In addition, two slices containing the rostral and middle parts of the CC body, respectively, were directly transferred into 4% paraformaldehyde in 1× phosphate-buffered saline (PBS, pH 7.4) to be fixed for immunofluorescent staining. The remaining slices were used for electrophysiology recordings. The CC fiber CAPs were evoked by constant current (0.1–0.5 mA, 40 μs in duration, 0.2 Hz) via a bipolar tungsten stimulating electrode. The recording electrodes, made from borosilicate glass capillaries (1.5/0.84 OD/ID, WPI, Sarasota, FL) and filled with 2 M NaCl (impedance 1–4 MΩ), were placed 1–1.5 mm away from the stimulating site. Signals were amplified through an Axopatch 1D amplifier (Molecular Devices, San Jose, CA) and a Dagan EX4-400 amplifier (Dagan Corp., Minneapolis, MN), filtered at 1 kHz, digitized at 5 kHz with Digidata 1440A interface (Molecular Devices), and recorded on a Dell computer with the pCLAMP 10.1 software (Molecular Devices).

### Western blot

The CC white matter tissues were dissected from the other half of the slices mentioned above in cold ACSF under an anatomic microscope. Then, the CC tissue was quickly transferred into Tissue Extraction Reagent 1 (FNN0071, Invitrogen, Camarillo, CA) with 1:1000 protease inhibitor (P-2714, Sigma) and homogenized. Protein concentration was measured by bicinchoninic acid assay (BCA assay). Routine electrophoresis was carried out using 10% sodium dodecyl sulfate-polyamide gel. Polyclonal rabbit anti-β-APP (1:600; AB5302, Millipore, Temecula, CA), monoclonal mouse anti-inducible nitric oxide synthase (iNOS; 1:500; AB49999, Abcam, Cambridge, MA), and rabbit anti-tumor necrosis factor alpha (TNF-α; 1:1000; AB66579, Abcam) were used to identify β-APP, iNOS, and TNF-α. Mouse anti-β-actin (1:10000; Sigma, A2228) was applied as a gel loading control. Immunoreactivity bands were detected using enhanced chemiluminescence and developed with autoradiography film.

### Immunocytochemistry

#### 1 Immunofluorescent staining

A total of 6 rats, two (a male and a female) from each group, were euthanized with isoflurane and transcardially perfused with saline followed by 4% paraformaldehyde in 1× PBS. The brains were removed and cryo-protected by gradient sucrose. Similarly, the two freshly fixed CC slices mentioned above underwent cryo-protection as well. Then, the coronal sections were cut at 10 μm thickness and mounted on slides immediately. About 10% of sections containing CC were selected to conduct immunofluorescent staining and intensity analysis. The sections were immunoblocked routinely and incubated with rabbit anti-ionizing calcium-binding adaptor protein-1 (Iba-1; 1:300; Wako Chemicals USA Inc., Richmond, VA), monoclonal rat anti-myelin basic protein (MBP; 1:200; Abcam), and polyclonal rabbit anti-neurofilament (NF; 1:400; Millipore) at room temperature overnight. Alexa Fluor 488-conjugated anti-rabbit antibodies (1:500; Molecular Probes, CA) were used for immunofluorescent visualization of microglia and nerve fibers. The control sections were stained in the same way without primary antibody.

#### 2 Proportional area and immunostaining intensity measurements

Microimages for proportional area measurement were acquired through a ×20 lens, and measurement was performed by using SlideBook (6.0.10; Intelligent Imaging Innovations Inc., Denver, CO). Iba-1 labeling was masked as the selected area at first, and the whole CC area in the scope field, excluding the area of lateral ventricle if any, was masked as the measured area that will be a denominator. Immunofluorescence intensity of MBP and NF was also collected and quantified using SlideBook. Likewise, images were acquired through a ×20 lens, the positive MBP or NF labeling was masked and the intensity was scaled, and the measured whole nerve bundle was masked as a denominator; then, those values were statistically analyzed and compared.

### Statistical analysis

FA and MD values were processed with one-way ANOVA with Tukey’s post-test of all pair of columns. One-way ANOVA analysis of the area under the curve (AUC), with Tukey’s post-test of all pair of columns, was used to test the differences between input-output (I/O) curves of CAPs recorded from the aforementioned three groups. Proportional area for Iba-1 staining, MBP, or NF immunoreactivity intensities and protein density of β-APP, iNOS, and TNF-α from the aforementioned three groups were also statistically compared with one-way ANOVA with Tukey’s post-test of all pair of columns. Statistics were processed by using GraphPad Prism 5 (GraphPad Software Inc., La Jolla, CA), and the significance level was indicated as *p* < 0.05 and *p* < 0.01, represented by “*” and “**” respectively.

## Results

### Disturbance of white matter linear integrity by LPS, partially rescued by minocycline

The measurement and analysis were focused on the rostral and middle CC regions, and fractional anisotropy was measured through the map (Fig. 1a), while the mean diffusivity was calculated by using the measured *λ*_L_ and *λ*_T_ parameters. FA reflects linear movements of water molecules, and MD, on the contrary, suggests a free diffusion of water and the extent of freedom [29]. Comparison between the saline, LPS, and LPS + minocycline groups by one-way ANOVA showed that water linear movement was significantly distorted (Fig. 1b; *p* < 0.05) by system LPS, and minocycline could reverse this distortion to an approximately significant level (*p* = 0.0657). ANOVA comparison of MD between the saline, LPS, and LPS + minocycline groups exhibited that LPS injection enhanced water diffusion almost to a significant level comparing to the saline injection did (Fig. 1c; *p* = 0.0570), while pre-delivery of minocycline appeared to attenuate this effect (Fig. 1c). In addition, a comparison of FA and MD values between male and female rats within the same group, namely in the saline, LPS, and LPS + minocycline groups, showed no evident difference.

### Declination of CC nerve fibers CAP by LPS, significantly inverted by minocycline

**Fig. 2:**
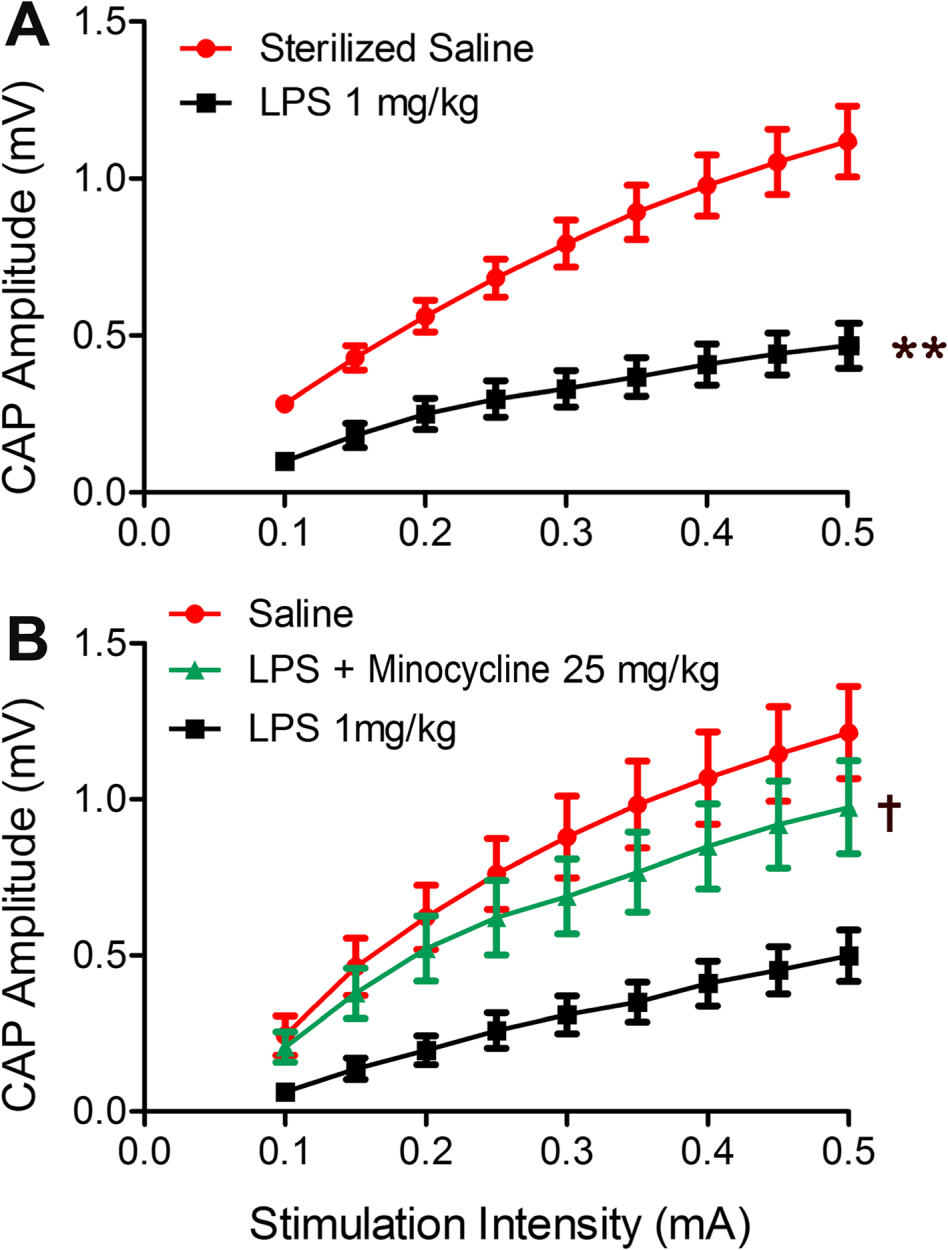
Declination of CC nerve fibers CAP by system LPS and inverted by minocycline. **a** LPS highly significantly downshifted the I/O curve of CC fibers CAP, revealed by AUC (areas under the curves) measurement and analysis, comparing to the I/O curve constructed in saline group (*N* = 4 in both saline and LPS groups; *p* < 0.01, shown as **). **b** Minocycline significantly reversed the system LPS-induced CAP declination also indicated by comparison of AUC between groups (*N* = 4 in both saline and LPS groups, *N* = 8 at LPS + minocycline cases; *p* < 0.05, shown as ^ϯ^), suggesting a prevention of minocycline for white matter tract from system LPS induced malfunction

CAPs were recorded in response to increment increase of stimulating intensities, and their amplitudes were measured and plotted as corresponding input-output (I/O) curve (Fig. 2). One-way ANOVA analysis of AUC displayed delivery of LPS highly significantly downshifted I/O curve versus the saline group (Fig. 2a; *p* < 0.01). This result suggests a functional impairment of white matter tract following system LPS. In addition, ANOVA statistical comparison of AUC values from the LPS group with that of the LPS + minocycline group showed minocycline significantly reversed the adverse effect of LPS on CAP (Fig. 2b; *p* < 0.05), reflecting protection of minocycline for white matter function against LPS-induced impairment.

### Minocycline reversed high densities of iNOS and TNF-α induced by LPS

**Fig. 3:**
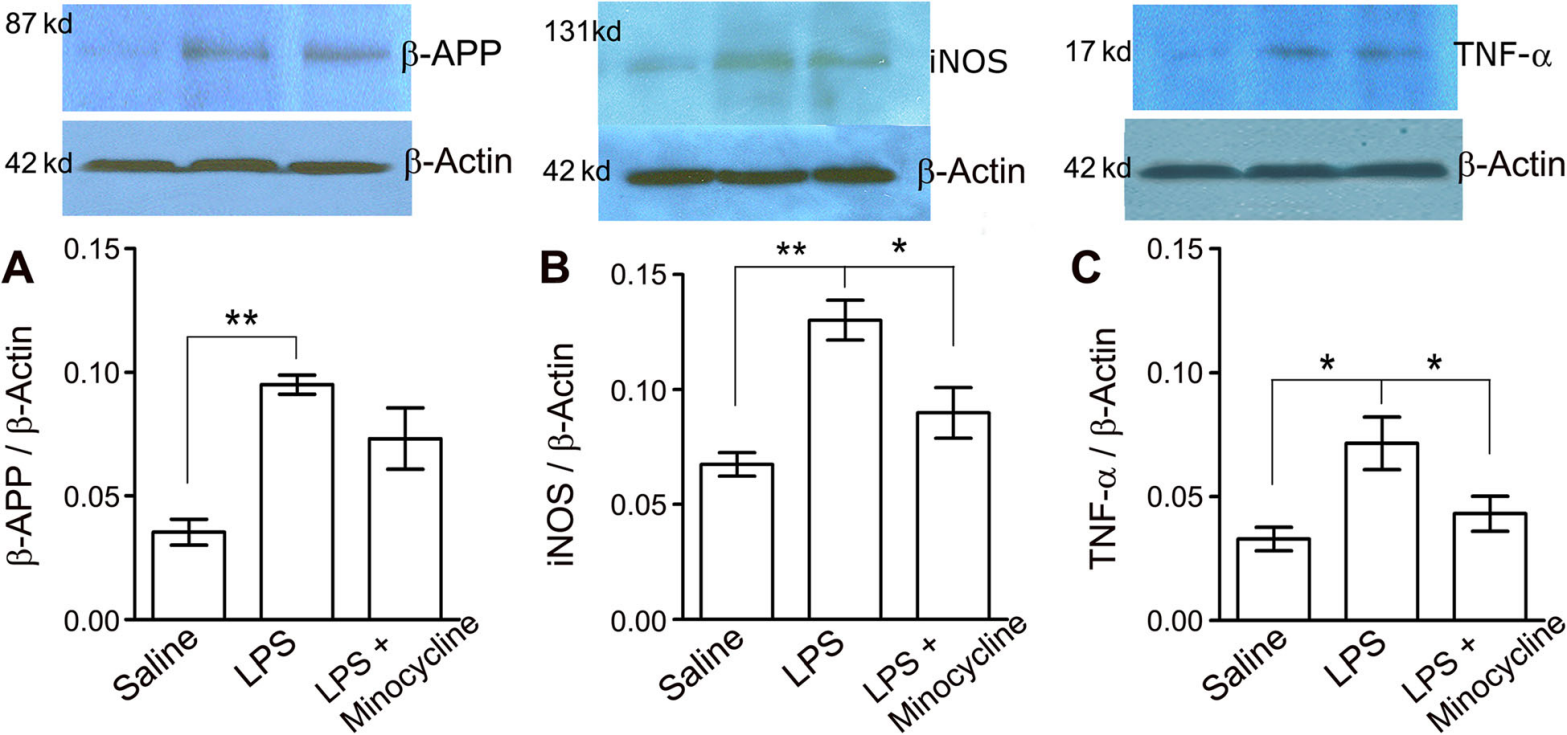
System LPS induced increment of β-APP, iNOS, and TNF-α, and minocycline inhibited their protein upgrading. **a** Accumulation of β-APP in the CC tissue reflected by a highly significantly higher level of protein in the LPS group than in the saline group (*N* = 4; *p* < 0.01, shown as **). Minocycline evidently reversed this accumulation but did not reach a significant level. **b** iNOS density in the CC of LPS group was highly significantly enhanced compared to that in the saline group (*N* = 4; *p* < 0.01, shown as **); also, minocycline significantly reversed high level of iNOS evoked by system LPS (*N* = 4; *p* < 0.05, shown as *). **c** Similarly, the CC TNF-α level in the LPS group was significantly higher than that in saline cases (*N* = 4; *p* < 0.05, shown as *); while minocycline significantly inhibited TNF-α upgrading (*N* = 4; *p* < 0.05, shown as *)

Protein densities of β-APP, iNOS, TNF-α, and β-actin were measured and normalized by corresponding β-actin level in each sample. The densities of β-APP and iNOS in CC tissues of the LPS injection group were significantly enhanced compared to that in the saline group (Fig. 3a, b; *p* < 0.01). The TNF-α level was significantly higher in the LPS group than in the saline group (Fig. 3c). In contrast, co-injection with minocycline significantly reversed the LPS evoked iNOS and TNF-α upregulation (Fig. 3b, c, *p* < 0.05), but not β-APP accumulation in the CC (Fig. 3a). Nonetheless, the impairment on fast axon transportation was ameliorated by co-delivery of minocycline.

### LPS activation of microglia and its attenuation by minocycline

**Fig. 4:**
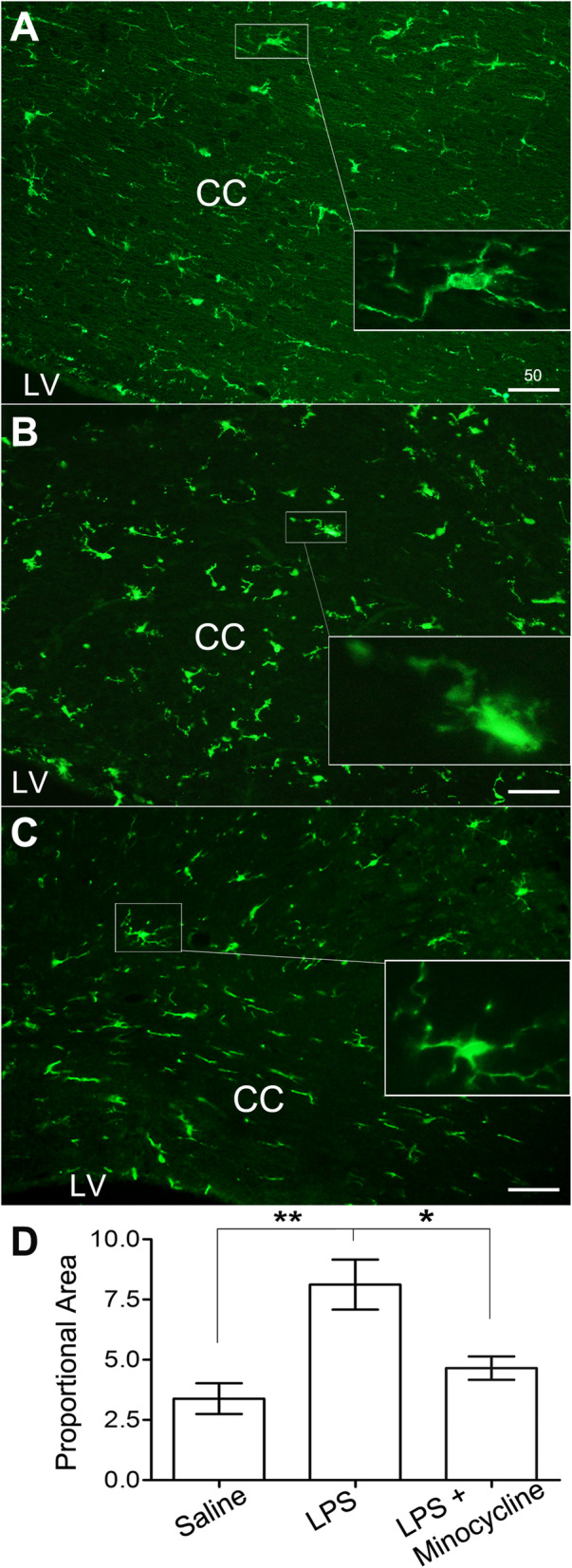
Activation of microglia by system LPS, attenuated by minocycline. **a** Resting microglia with thin somata and delicate pseudopodia were observed in the CC of the saline group as shown in the inset. **b** Majority of Iba-1-positive microglia in LPS-treated CC were hyper-ramified with hypertrophic somata or showing amoeba-like morphology as viewed in the inset. **c** Hypertrophic somata and/or pseudopodia were also viewed in the minocycline co-injected group (see the inset); whereas, amoeba-like microglia was almost vanished. **d** Statistical processing of proportional area data displays that system LPS has highly significantly expanded proportional area (*N* = 3 in both saline and LPS groups; *p* < 0.01, shown as **), while minocycline significantly reversed proportional area values compared to that in the LPS group (*N* = 6 in LPS + minocycline cases; *p* < 0.05, shown as *). Scale bars in **a**–**c** are 50 μm

Resting microglia with thin somata and delicate pseudopodia were observed in the CC of the saline group as revealed by Iba-1 immunofluorescent staining (Fig. 4a and inset). A large number of Iba-1-positive microglia were hyper-ramified with hypertrophic somata or even amoeba like (Fig. 4b and inset) in the LPS group. When minocycline was co-administrated with LPS, microglia with amoeba-like appearance became much fewer, and hypertrophic pseudopodia and/or mild hypertrophic soma were also observed (Fig. 4c and inset). Proportional area values [30] were calculated by normalizing the selected area with the whole measured area. ANOVA analysis indicated a highly significant morphological change evoked by system LPS compared to that from the saline group (Fig. 4d; *p* < 0.01). Such morphological changes were significantly attenuated by co-administration of minocycline (Fig. 4d; *p* < 0.05).

### Activation of microglia on white matter fiber integrity

**Fig. 5:**
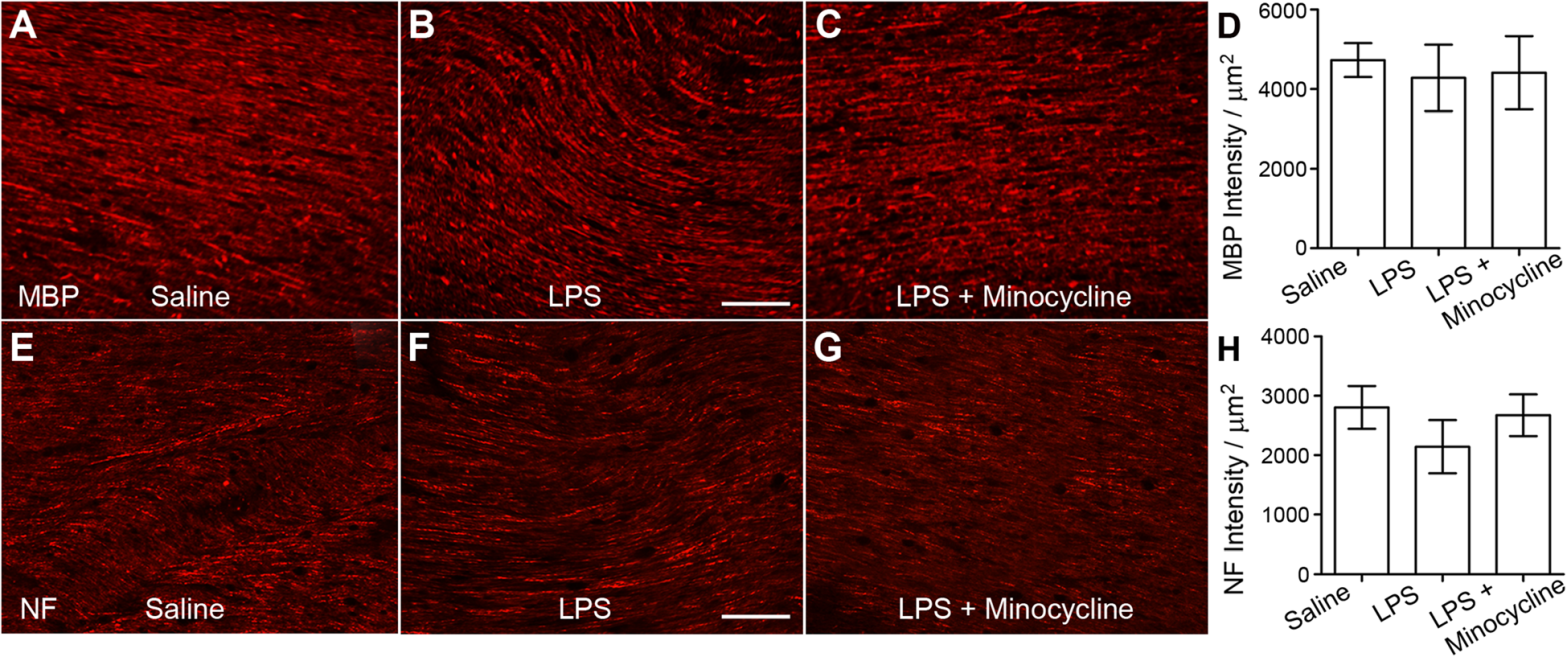
In situ measurement of MBP and NF protein densities showed no significant change. **a**–**c** MBP immunostained myelinated nerve fibers in saline cases (**a**), LPS group (**b**), and LPS + minocycline animals (**c**). **d** Statistical comparison of measured MBP immunofluorescence intensity exhibits no apparent difference between the saline, LPS, and LPS + minocycline groups (*N* = 3 in the saline and LPS groups, *N* = 4 in the LPS + minocycline rats). e–g NF immunoreactivity of CC nerve fibers in the saline (**e**), system LPS (**f**), and LPS + minocycline groups (**g**). **h** Statistical process of measured NF immunofluorescence intensity shows no significant difference between the 3 groups (*N* = 3 in the saline and LPS groups, *N* = 4 in the LPS + minocycline rats); however, NF intensity of immunofluorescent labeling in the LPS group was obviously lower than that in the saline and LPS + minocycline groups. Scale bars for a–c and e–g are 50 μm

Densities of MBP and NF reflect white matter tract integrity. The MBP primarily signifies myelinated nerve fibers, and the NF reflects the integrity of both myelinated and unmyelinated fibers [31, 32]. To examine if injection of LPS injures white matter fibers, we measured the intensity of immunofluorescence that labels MBP (Fig. 5a–c) and NF (Fig.5e–g), digitized their densities, and then normalized by the whole area of measured nerve fibers in the CC. Statistical analyses showed no significant difference between the saline, LPS, and LPS + minocycline groups (Fig. 5d, h), indicating no significant damage on myelinated nerve fibers after administration of LPS. However, the intensity of NF labeling in the LPS group did exhibit a somewhat decrease which was reversed by minocycline (Fig. 5h), implying some adverse effect of microglia activation on NF transportation and assembly.

## Discussion

Using LPS to induce microglia activation ex vivo, we have previously demonstrated that conditioned microglia activation in rat CC resulted in white matter tract malfunction as illustrated by a reduction of CAP magnitude and an impairment of fast axon transport reflected by accumulation of β-APP [17]. The extent of microglia activation was correlated to the alterations of CAP and fast axon transport of β-APP. Based on our previous experimental results [17] and the findings by others [33, 34], we further investigated and validated, using an in vivo semi-quantification test, the extent of white matter tract abnormalities with the states of microglia activation, focusing on the CC region. We also examined whether the LPS-associated white matter tract abnormalities can be ameliorated or blocked by an anti-inflammatory agent minocycline. Our results showed that microglia activation induced by systemic administration of LPS-produced white matter tract malfunction in the CC and minocycline, a semisynthetic tetracycline derivative, attenuated LPS-induced microglia activation and resultant white matter abnormalities in the CC.

Increasing evidence indicate that neuroinflammation provoked by microglia activation plays an important role in the pathogenesis of neurological disorders [6–12]. Using LPS to mimic an etiological factor to induce microglial activation in many neurological disorders is widely used in animal studies [35–37]. It has been shown that LPS at a dose of 1 mg/kg/day through i.p. delivery was not possible to impair the blood-brain barrier and enter the brain freely [35, 36]. In contrast, three consecutive deliveries were able to effectively activate microglia, and full activation was observed at the third day after systemic delivery [33, 34]. Using a 3-day delivery protocol, we did observe microglia activation and functional impairment in the CC. Our results were in a full agreement with those observations made by others [33, 34].

In a recent study, the authors reported that systemic delivery of LPS for 3 days resulted in a significant malfunction of the hippocampus and the CC nerve fibers with robust microglia activation in both brain regions [33]. Three days post-LPS delivery, hippocampal function largely recovered with a reduced microglia activation, while the CC nerve fibers malfunction exacerbated [33]. Their results were in consistent with a prior report that activation of microglia was detected 8–24 h after systemic LPS delivery, and explicit morphological change was visualized at the 3rd day following 1 or 2.5 mg/kg LPS injection [34]. Noteworthy, comparison of microglia activation levels in the CC with that in the hippocampus at that time point showed significantly higher in the CC than that in the hippocampus [33].

In the present work, we adopted a similar procedure to evoke microglia activation as employed by other researchers [33, 34] and examined potential alterations on white matter integrity and function in the CC. We observed that systemic administration of LPS induced microglia activation as determined by labeling Iba-1-positive microglial cells and the morphological changes as measured and assessed using a proportional area comparison [30, 38]. LPS-induced microglia activation was supported by elevated expression levels of inflammatory markers, such as iNOS and TNF-α. The observed changes associated with LPS-stimulated microglia activation include (1) an interruption of the CC fibers linear integrity detected by diffusion tensor magnetic resonance imaging (DT-MRI), which can map movements of water molecules and image linear integrity of the white matter tract [29]; (2) a reduction on the magnitude of electrical evoked axonal CAPs, implying for a functional change of the white matter tracts [17, 33]; and (3) an accumulation of β-APP (which travels along axons through fast axon transportation), a well-accepted marker for white matter tracts injury [39, 40]. These alterations were partially rescued or significantly reversed by administration of minocycline. Besides, we analyzed the expression levels of myelin basic protein (MBP) and neurofilament (NF) by immunostaining and found no significant change on MBP and slight reduction of NF, suggesting that degeneration or demyelination of nerve fibers was not ensued in the time course adopted in this study.

How systemically administered LPS enters the brain is not fully understood. A study using radiocarbon-labeled LPS to examine how the LPS passes the blood-brain barrier (BBB) demonstrated that binding to LPS receptors on the endothelium membrane is probably the predominant pathway through which the LPS enters the brain parenchyma [35]. The LPS might bind to endothelial CD14 (cluster of differentiation 14) and/or Toll-like receptors (TLR) 4 and 2 to cross the BBB, as expression levels of endothelial CD14 mRNAs were upregulated shortly after LPS injection and overexpression of TLR mRNAs lasted for a longer time [35]. Epithelial cells of the choroid plexus also express CD14, and their mRNAs in the epithelium were significantly elevated for a much longer time than in BBB endothelium following system LPS treatment [41]. Moreover, a significant increment of pro-inflammatory immune cells crossing choroid plexus into the cerebrospinal fluid (CSF) is observed after system LPS [42, 43]. These reports imply the first delivery of LPS might ensue a little amount of LPS into the brain parenchyma. This implication was supported by experimental results that the concentration of LPS in the brain parenchyma was only 0.025% of the circulating LPS following a single injection of doses from 0.1 to 5 mg/kg [36]. In addition, the first cytokine released into the circulation after stimulation of the immune system with LPS was TNF-α [44], suggesting that TNF-α might be the first pro-inflammatory cytokine entering the brain from the circulation following systemic LPS. However, a study in rats using isotope-labeled TNF-α to monitor the rate of its crossing the BBB in both physiological condition and systemic LPS unveiled no significant change in up-taking rate for isotope-labeled TNF-α between 4 and 24 h after administration (i.p.) of LPS [45]. TNF receptors 1 and 2 on BBB endothelium transport TNF-α crossing BBB whenever the BBB is intact [46, 47] and their mRNA expression levels in the endothelium were upregulated following LPS treatment, but their receptor proteins exhibited no significant increase [45]. This paradox may explain why no significant change in up-taking rate for isotope-labeled TNF-α was detected after administration (i.p.) of LPS [45].

In light of the aforementioned studies, we assume that (1) the first LPS injection might prime resident microglia through a limited amount of LPS and TNF-α entrance into the brain from circulation. The microglia in the CC immediately adjacent to the ventricles might be activated in an earlier time fashion, considering the pro-inflammatory monocytes, granulocytes, and lymphocytes in the CSF are significantly increased [42, 43]. (2) The second injection of LPS might activate most of the microglia and the third delivery further challenged microglia, leading to the production of a significant amount of pro-inflammatory cytokines including TNF-α, as reflected by our western blot results. (3) Microglia were eventually agitated to become neurotoxic because upregulated iNOS expression, as revealed by our western blot results, is a sign of detrimental microglia activation [48, 49]. In this case, the observed malfunction of the white matter tract was most likely due to activated microglia since our proportional area measurement of Iba-1 immunoreactivity [30] displayed a positive correlation between microglia activation and severity of white matter malfunction. The direct adverse effect of LPS and/or circulating TNF-α on white matter tract, if any, could be ignorable.

It has been shown that systemic administration of LPS and overactivated microglia affected both grey matter in the hippocampus and white matter in the CC, with a prolonged adverse effect on the CC [33]. The mechanism underlying such a difference is still unclear. Based on our recent findings that microglia pseudopodia directly contact node of Ranvier or node-like sodium channel clusters on the CC nerve fibers, it might be possible that activation of microglia changed the contacting ratio and pattern between pseudopodia and node-like structures [50]. In addition, a large CC area abutting to CSF in the ventricles may facilitate interaction between pro-inflammatory substances in the CSF and the microglia on the CC border [42, 43]. Nevertheless, the present in vivo studies demonstrated that activation of microglia had a noxious effect in the CC. Such an effect might be ensued as a significant increment of iNOS, an established sign for detrimental microglia activation [48, 49]. In parallel, malfunction of the CC nerve fibers was reflected by interruption of fiber bundle linear integrity as detected by DT-MIR, reduction of CC nerve fibers CAP magnitude, and accumulation (decelerated transportation) of β-APP through axons in the CC.

## Conclusions

Systemic administration of LPS induced microglia activation in the CC of rats, resulting in morphological and physiological alterations that were ameliorated or reverted by minocycline, a well-characterized anti-inflammatory agent. As white matter injury has been detected in Alzheimer’s disease [51–53], Parkinson’s disease [54, 55], and HIV-associated dementia [56–58], the LPS-induced microglia activation and resultant white matter abnormalities may represent an exemplary mechanism for the pathogenesis of neurodegenerative disorders. Thus, the amelioration or revert of activated microglia-induced white matter abnormalities by minocycline implies a therapeutic potential for the aforementioned neurological diseases.

## Data Availability

The data that support the findings of this study are available from corresponding authors upon reasonable request.

## Abbreviations

ACSF: Artificial cerebrospinal fluid
BBB: Blood-brain barrier
CAP: Compound action potential
CC: Corpus callosum
CNS: Central nervous system
CSF: Cerebrospinal fluid
FA: Fractional anisotropy
HIV-1: Human immunodeficiency virus type 1
i.p.: Intraperitoneally
LPS: Lipopolysaccharide
MBP: Myelin basic protein
MD: Mean diffusivity
NF: Neurofilament
ROIs: Regions-of-interest
TLR: Toll-like receptors
